# miR-186 Inhibits Liver Cancer Stem Cells Expansion *via* Targeting PTPN11

**DOI:** 10.3389/fonc.2021.632976

**Published:** 2021-03-18

**Authors:** Haochen Yao, Ziting Yang, Yan Lou, Juanjuan Huang, Pinghua Yang, Weiqi Jiang, Shuai Chen

**Affiliations:** ^1^Department of Emergency Surgery, The First Hospital of Jilin University, Changchun, China; ^2^Department of Pathogenobiology, The Key Laboratory of Zoonosis, Chinese Ministry of Education, College of Basic Medical Science, Jilin University, Changchun, China; ^3^Department of Emergency, The 964th Hospital of the Chinese People’s Liberation Army, Changchun, China; ^4^Department of Nephrology, The Second Hospital of Jilin University, Changchun, China; ^5^Department of Hepatic Surgery, Third Affiliated Hospital of Second Military Medical University, Shanghai, China

**Keywords:** hepatocellular carcinoma, cancer stem cells, miR-186, PTPN11, transcatheter arterial chemoembolization (TACE)

## Abstract

MicroRNAs (miRNAs) participated in the regulation of tumorigenesis, progression, metastasis, recurrence and chemo-resistance of cancers. However, the potential function of miRNAs in cancer stem cells (CSCs) or tumor-initiating cells (T-ICs) was not clearly elucidated. In the present study, we found that miR-186 expression was reduced in liver CSCs. Functional studies showed that miR-186 knockdown facilitated liver CSCs self-renewal and tumorigenesis. Conversely, forced miR-186 expression suppressed liver CSCs self-renewal and tumorigenesis. Mechanically, miR-186 downregulated PTPN11 *via* binding to its 3’-UTR in liver CSCs. The correlation of miR-186 and PTPN11 was confirmed in Hepatocellular carcinoma (HCC) patients’ tissues. Further study showed that interference of PTPN11 can abolished the discrepancy between miR-186 mimic and control HCC cells in self-renewal and the proportion of CSCs. Additionally, we found that miR-186 overexpression HCC cells were more sensitive to cisplatin treatment. Clinical cohort analysis showed that HCC patients with high miR-186 were benefited more from transcatheter arterial chemoembolization (TACE) treatment. In conclusion, our study demonstrates a new regulation mechanism of liver CSCs, a new target for HCC, and a biomarker for postoperative TACE.

## Introduction

Hepatocellular carcinoma (HCC) is one of the most common malignant cancers in the world (1). The incidence of HCC increases yearly and more than half of new cases are in China. Most HCC patients are diagnosed at advanced stage and lost their best surgery chance (2–4). At present, transcatheter arterial chemoembolization (TACE), targeted drug and immunotherapy are the main treatment for advanced HCC patients, but these treatments are unsatisfactory (5–7). So, it is urgent to explore the underlying mechanism of HCC initiation and progression.

MicroRNAs (miRNAs) contain about 22 nucleotides and are involved in the regulation of biological process and pathogenesis (8). miRNAs work as oncogenes or tumor suppressors in different tumors. Accumulating evidence shows that miRNAs can used as therapeutically targets and prognosis biomarkers (9, 10). miRNA class is a significant class of post-transcriptional gene expression regulator to control the self-renewal and differentiation properties of cancer stem cell (11). miR-186 is a newly discovered miRNA whose role in biological process and pathogenesis is not fully studied. Previous studies found that miR-186 promotes the apoptosis of glioma U87 cells by down-regulating the expression of Smad6 (12). miR-186 negatively regulate FOXD1 to promote the proliferation, metastasis and radio-resistance of nasopharyngeal carcinoma cells (13). However, the function of miR-186 in liver CSCs remains unclear.

Cancer stem cells (CSCs) are subpopulation of cancer cells which have the ability of infinite proliferation, self-renewal, tumorigenesis and chemo-resistance (14). CD133, CD90, CD24 and EpCAM are well-accepted liver CSCs markers and can be used to identify liver CSCs (15–18). CD24 can drive self-renewal and tumorigenesis of liver CSCs *via* STAT3-mediated Nanog pathway (17). HCC patients with high proportion of liver CSCs indicates the poor prognosis (19). Therefore, identification of the underlying mechanisms governing liver CSCs expansion may lead to the discovery of promising therapeutic strategies for HCC patients.

In this study, we first find that miR-186 is reduced in liver CSCs. Next, by using loss-of-function analysis and gain-of-function analysis in liver CSCs, we demonstrate that miR-186 inhibits the self-renewal capacity and tumorigenicity of liver CSCs. Further mechanism study reveals that miR-186 directly targets PTPN11 by binding to its 3’UTR. Interestingly, we find that miR-186 overexpression HCC cells are sensitive to cisplatin treatment. Clinical cohort analysis demonstrates that miR-186 high HCC patients are benefited from TACE treatment. Altogether, we discover that miR-186 suppress the expansion of liver CSCs *via* interacting with PTPN11.

## Materials and Methods

### Cell Lines and Cell Culture

Patient-derived primary HCC cultures of tumor cells were obtained from fresh tumor specimens of HCC patients as previously described. The human primary hepatoma cells were isolated by collagenase perfusion and centrifugation. Briefly, the HCC tissues were washed several times in pre-cooled sterile PBS buffer containing double antibodies to remove blood and connective tissue; GBSS mixed enzyme solution was used for digestion. The cells were centrifuged, and the supernatant was discarded. Cells viability and counting were performed using trypanosoma blue staining with cell filtrate, and cultured in a bottle containing complete medium heavy suspension at 37°C and 5% CO_2_ environment culture. After the cell adheres to the wall and the cell morphology was identified.

The HCC cell lines Huh7 and Hep3B was purchased form the Chinese Academy of Sciences (Shanghai, China). The HCC cells were cultured with Dulbecco’s modified Eagle’s medium (DMEM) supplemented with 10% fetal bovine serum (FBS) and 2 mM L-glutamine, and 25 µg/ml of gentamicin and maintained at 37°C in 5% CO_2_ incubator. The cultured cells were digested with 0.5% trypsin and moved to a new plate twice a week. miR-816 mimic (sense, 5′‐CAAAGAAUUCUCCUUUUGGGCU‐3′; antisense, 5′‐CCCAAAAGGAGAAUUCUUUGUU‐3′), or miR-186 sponge (5′‐AGCCCAAAAGGAGAAUUCUUUG‐3′) lentivirus and their control lentivirus were purchased from Shanghai GenePharma (Shanghai, China).

### Patients and Samples

The HCC and corresponding peritumoral tissues were collected from surgical resections of patients without preoperative treatment at Eastern Hepatobiliary Surgery Hospital (EHBH, Shanghai, China). A total of 60 patients received adjuvant TACE therapy after surgery for primary HCC at EHBH from 2010 to 2015 were included in Cohort 1. Detailed clinicopathological features of these patients are described in **Supplementary Table 1**. Patient informed consent was obtained and the procedure of human sample collection was approved by the Ethics Committee of EHBH.

### Flow Cytometry Analysis

For CD133^+^ and CD90^+^ cells sorting, primary HCC patients’ cells and HCC cells were incubated with the primary anti–CD133 (Cat. no. 372806, Biolegend, Inc., San Diego, CA) or anti-CD90 (Cat. no. ab225; Abcam, USA) for 30 min at room temperature. The cells were then subjected to flow cytometry using a MoFlo XDP cell sorter from Beckman Coulter (Indianapolis, IN, USA) according to the manufacturer’s instructions. The sorted cells from three independent experiments were subjected to Real-time PCR assay.

### Spheroid Formation Assay

HCC cells were cultured in a 96-well ultra-low attachment (300 cells per well) and cultured in DMEM/F12 (Gibco) media, supplemented with 1% FBS, 20 ng/ml bFGF and 20 ng/ml EGF for seven days. The total number of spheres was counted under the microscope (Olympus).

### *In Vitro* Limiting Dilution Assay

Various numbers of HCC cells (2, 4, 8, 16, 32, and 64 cells per well) were seeded into 96-well ultra-low attachment and cultured in DMEM/F12 (Gibco) supplemented with 1% FBS, 20 ng/ml bFGF and 20 ng/ml EGF for seven days. The CSC proportions were analyzed using Poisson distribution statistics and the L-Calc Version 1.1 software program (Stem Cell Technologies, Inc., Vancouver, Canada) as previously described (20).

### *In Vivo* Limiting Dilution Assay

For the *in vivo* limiting dilution assay, different concentrations of HCC cells (1 × 10^3^, 5 × 10^3^, 1 × 10^4^, and 5 × 10^4^) were mixed with equal volume of Matrigel (1:1) and inoculated subcutaneously to NOD-SCID mice (n=6). Tumor formation was observed two months later.

### Real-Time PCR

For detection of mature miRNA, total RNA was subjected to reverse transcription using a TaqMan MicroRNA Reverse Transcription Kit (Applied Biosystems). qRT-PCR analysis of miR-186 expression was carried out using TaqMan MicroRNA assay kits (Applied Biosystems). Results were normalized to U6 snRNA using the comparative threshold cycle (Ct) method.

The total RNA was extracted by using Trizol reagent (Invitrogen, 15596-018). Total cDNAs were synthesized by ThermoScript TM RT-PCR system (Invitrogen, 11146-057). The total mRNA amount present in the cells was measured by RT-PCR using the ABI PRISM 7300 sequence detector (Applied Biosystems). PCR conditions included 1 cycle at 94°C for 5 min, followed by up to 40 cycles of 94°C for 15 s (denaturation), 60°C for 30 s (annealing) and 72°C for 30 s (extension). The sequences of primers used was listed in **Supplementary Table 2**.

### Western Blotting Assay

Thirty micrograms of proteins were subjected to sodium dodecyl sulfate polyacrylamide gel electrophoresis and then transferred to nitrocellulose membrane. The membrane was blocked with 5% non-fat milk and incubated with the primary antibody overnight. The protein band, specifically bound to the primary antibody, was detected using an IRDye 800CW-conjugated secondary antibody and LI-COR imaging system (LI-COR Biosciences, Lincoln, NE, USA). The primary antibodies used were listed in **Supplementary Table 3**.

### Luciferase Reporter Assay

A 300-bp fragment of the PTPN11 3’UTR containing the conserved miR-186-binding sites was inserted into a luciferase reporter plasmid. The PTPN11 3’UTR mutant luciferase plasmid contained changes in potential miR-186-binding base sequence “UUCUUU” to “GCGAGC”. Then the 300-bp fragment of PTPN11 mutant 3’UTR fragment was inserted into a luciferase reporter plasmid. miR-186 mimic or miR-186 sponge and their control hepatoma cells were transfected with PTPN11 WT or PTPN11 mutant 3’UTR plasmids. The luciferase activity was measured using a Synergy 2 Multidetection Microplate Reader (BioTek Instruments, Inc.). The data were normalized for transfection efficiency by dividing firefly luciferase activity by Renillaluciferase activity.

### Apoptosis Assay

miR-186 mimic or miR-186 sponge and their control HCC cells were treated with cisplatin (4 μg/L) for 48 h, followed by staining with Annexin V and 7-AAD for 15 min at room temperature in the dark. Apoptotic cells were determined by an Annexin VFITC Apoptosis Detection Kit I (BD Pharmingen, San Diego, CA) and detected by flow cytometry according to the manufacturer’s instructions.

### Statistical Analysis

GraphPad Prism (GraphPad Software, Inc. La Jolla, USA) was used for all statistical analyses. Statistical analysis was carried out using t test or Bonferroni Multiple Comparisons Test: *p<0.05. A p value of less than 0.05 was considered significant.

## Results

### miR-186 Is Downregulated in Liver Cancer Stem Cells

**Figure 1:**
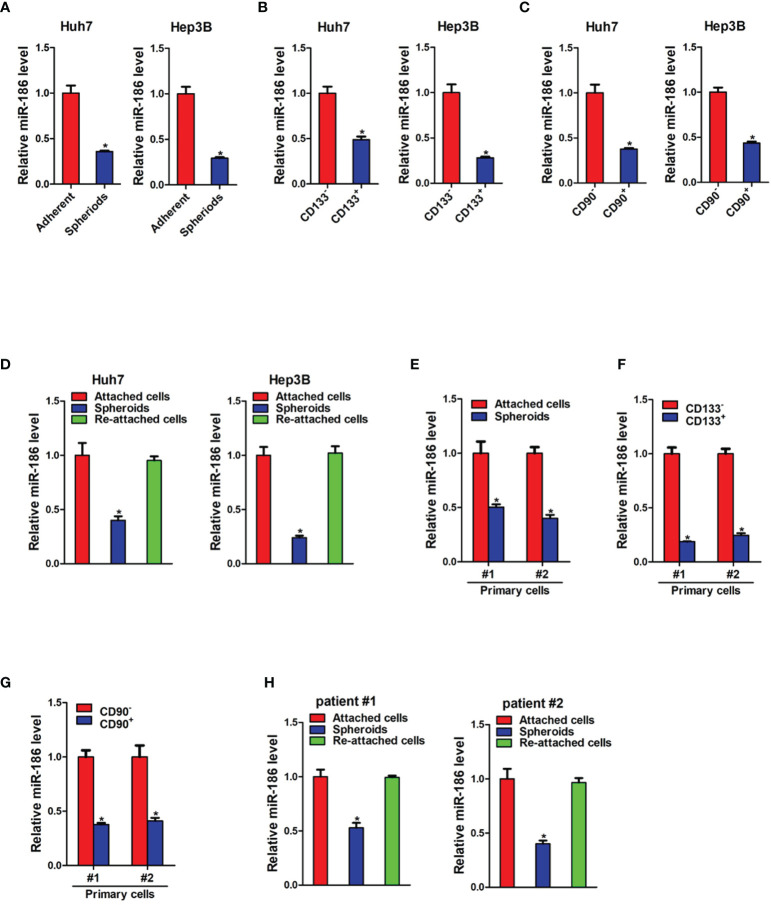
miR-186 is downregulated in liver CSCs. **(A)** The expression of miR-186 in hepatoma cells with low adhesion culture and adherent culture was detected by real-time PCR. **(B)** The expression of miR-186 in CD133^+^ hepatoma cell lines and CD133^-^ hepatoma cell lines was detected by real-time PCR. **(C)** The expression of miR-186 in CD90^+^ hepatoma cell lines and CD90^-^ hepatoma cell lines was detected by real-time PCR. **(D)** The expression of miR-186 in adherent culture, low adhesion culture and re-adherent culture hepatoma cells was determined by real-time PCR. **(E)** The expression of miR-186 in primary hepatoma cells with low adhesion culture and adherent culture was checked by real-time PCR. **(F)** The expression of miR-186 in CD133^+^ primary hepatoma cell lines and CD133^-^ primary hepatoma cell lines was checked by real-time PCR. **(G)** The expression of miR-186 in CD90^+^ primary hepatoma cell lines and CD90^-^ primary hepatoma cell lines was checked by real-time PCR. **(H)** The expression of miR-186 in adherent culture, low adhesion culture and re-adherent culture primary hepatoma cells was checked by real-time PCR. "*" means p<0.05. There was a statistical difference.

Low adhesion spheroid formation is a commonly used method for enrichment of cancer stem cells. As shown in **Figure 1A**, miR-186 expression was dramatically downregulated in hepatoma cell spheroids than in adherent cells. We also isolated CD133 positive and CD90 positive cells from adherent hepatoma cells by using flow cytometry. The expression of miR-186 was reduced in CD133 positive cells or CD90 positive cells compared with their negative control cells (**Figures 1B, C**). Notably, miR-186 level was increased to origin level when the spheres were reattached (**Figure 1D**). Next, miR-186 expression was verified in primary hepatoma cells. Compared with adherent cells, miR-186 expression was decreased in HCC spheres derived from human primary HCC cells (**Figure 1E**). Consistently, miR-186 levels were downregulated in sorted CD133 positive or CD90 positive primary HCC cells (**Figures 1F, G**). Interestingly, miR-186 level could be restored during reattachment in parallel with the differentiation (**Figure 1H**). Taken together, the results demonstrated that miR-186 expression was reduced in liver CSCs.

Next, we measured miR-186 expression in human HCC tissues. As shown in **Supplementary Figure 1A**, miR-186 expression was significantly reduced in HCC tumor tissues compared with the paired non-tumorous tissues. We also checked the expression level of miR-186 in normal hepatocytes, HCC cells and liver CSCs. The results showed that the expression level of miR-186 was much lower in liver CSCs than HCC cells and normal hepatocytes (**Supplementary Figure 1B**). Increasing evidence showed that DNA methylation or histone acetylation is a major cause of tumor suppressor silencing in cancer, HCC cells were treated with different concentrations of DNA methyltransferase inhibitor 5-aza-2’-deoxycytidine (5-AZA) or TSA to determine whether miR-186 downregulation was associated with DNA methylation or histone acetylation in HCC. Treatment of CCA cells with TSA did not change the level of miR-186, while 5-AZA led to a restoration of miR-186 expression, suggesting that there was a negative correlation between DNA methylation and miR-186 expression (**Supplementary Figure 1C**).

### miR-186 Knockdown Promotes Liver Cancer Stem Cell Expansion

**Figure 2:**
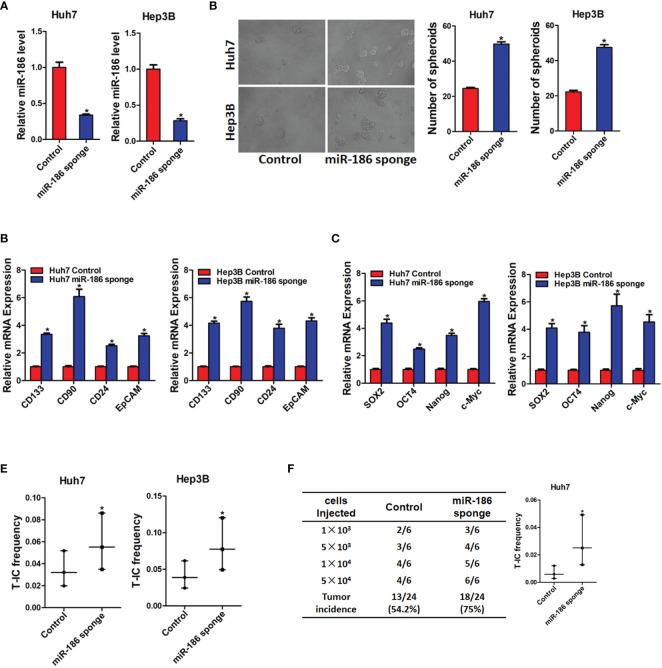
Interference of miR-186 promotes liver CSCs expansion. **(A)** Huh7 and Hep3B were infected with miR-186 sponge virus. The interference effect of miR-186 sponge was checked by real-time PCR analysis. **(B)** The expression of CSC markers CD133, CD90, CD24, and EpCAM in miR-186 knockdown cells and control HCC cells was determined by real-time PCR analysis. **(C)** The expression of transcription factors Sox2, Oct4, Nanog, and c-Myc in miR-186 knockdown cells and control HCC cells was detected by real-time PCR analysis. **(D)** miR-186 interference cells and control HCC cells were seeded in low adhesion 96-well plate, and spheroids were counted and photographed after 7 days. **(E)** Gradient concentrations of miR-186 knockdown cells and control HCC cells were seeded in low adhesion 96-well plate, and spheroids formation in each group was observed 7 days later, and the proportion of CSCs was counted and calculated. **(F)** Different concentrations of Huh7 miR-186 knockdown cells and control HCC cells were mixed with equal volume of Matrigel and inoculated subcutaneously to NOD-SCID mice (n=6). Tumor formation was observed two months later. "*" means p<0.05. There was a statistical difference.

Next, we want to explore the potential role of miR-186 in liver CSCs. HCC cell lines Huh7 and Hep3B were infected with miR-186 sponge virus or control virus. The knockdown effect was confirmed by real-time PCR analysis (**Figure 2A**). As shown in **Figure 2B**, the expression of CSCs markers CD133, CD90, CD24, and EpCAM were significantly increased in miR-186 knockdown HCC cells compared with control HCC cells. Increasing evidence shows that several transcription factors such as Sox2, Oct4, Nanog and c-Myc participate in the regulation of liver CSCs (21). Our data found that the expression of Sox2, Oct4, Nanog and c-Myc were also upnregulated in miR-186 interference HCC cells compared with control HCC cells (**Figure 2C**). The ability of self-renewal is characteristic for liver CSCs. We found that the spheroids-forming ability was markedly enhanced in miR-186 knockdown HCC cells compared with control cells under low adhesion culturing circumstance (**Figure 2D**). Moreover, *In vitro* limiting dilution assay showed that the proportion of CSCs were dramatically increased in miR-186 interference HCC cells (**Figure 2E**). *In vivo* limiting dilution assay of NOD-SCD mice showed that the tumorigenicity ability was significantly upregulated in miR-186 knockdown Huh7 cells compared with the control hepatoma cells (**Figure 2F**).

### miR-186 Overexpression Inhibits Liver CSCs Expansion

**Figure 3:**
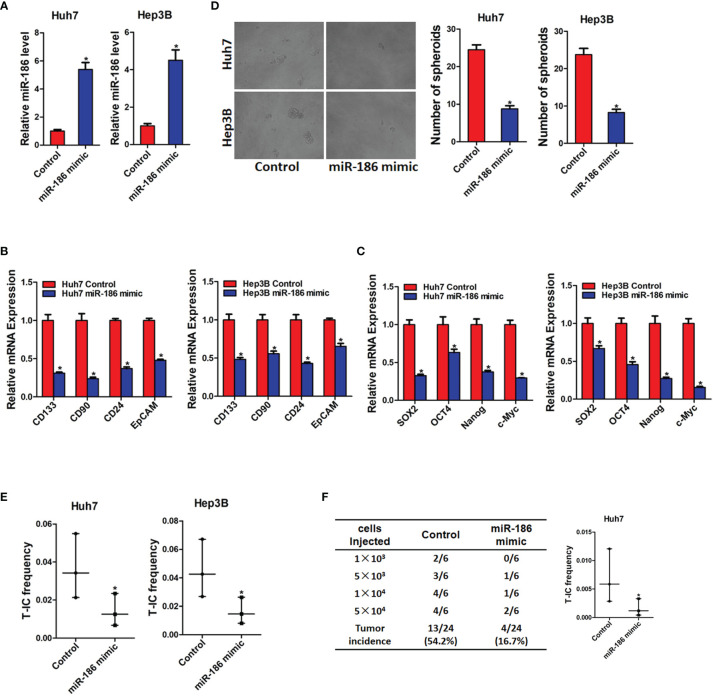
Overexpression of miR-186 suppresses liver CSCs expansion. **(A)** Huh7 and Hep3B were infected with miR-186 overexpression virus. The overexpression effect of miR-186 mimic was checked by real-time PCR analysis. **(B)** The expression of CSC markers CD133, CD90, CD24, and EpCAM in miR-186 overexpression cells and control HCC cells was determined by real-time PCR analysis. **(C)** The expression of transcription factors Sox2, Oct4, Nanog, and c-Myc in miR-186 overexpression cells and control HCC cells was detected by real-time PCR analysis. **(D)** miR-186 overexpression cells and control HCC cells were seeded in low adhesion 96-well plate, and spheroids were counted and photographed after 7 days. **(E)** Gradient concentrations of miR-186 overexpression cells and control HCC cells were seeded in low adhesion 96-well plate, and spheroids formation in each group was observed 7 days later, and the proportion of CSCs was counted and calculated. **(F)** Different concentrations of Huh7 miR-186 overexpression cells and control HCC cells were mixed with equal volume of Matrigel and inoculated subcutaneously to NOD-SCID mice (n=6). Tumor formation was observed two months later. "*" means p<0.05. There was a statistical difference.

Next, miR-186 overexpression HCC cells were used. HCC cell lines Huh7 and Hep3B were infected with miR-186 mimic virus or control virus. The overexpression effect was confirmed by real-time PCR analysis (**Figure 3A**). As shown in **Figure 3B**, the expression of CSCs markers CD133, CD90, CD24, and EpCAM were significantly decreased in miR-186 overexpression HCC cells compared with control HCC cells. Our data also found that the expression of Sox2, Oct4, Nanog, and c-Myc were downregulated in miR-186 overexpression HCC cells compared with control HCC cells (**Figure 3C**). The ability of self-renewal is characteristic for liver CSCs. We found that the spheroids-forming ability was markedly impaired in miR-186 overexpression HCC cells compared with control cells under low adhesion culturing circumstance (**Figure 3D**). Moreover, *In vitro* limiting dilution assay showed that the proportion of CSCs were dramatically decreased in miR-186 overexpression HCC cells (**Figure 3E**). *In vivo* limiting dilution assay of NOD-SCD mice showed that the tumorigenicity ability was significantly downregulated in miR-186 overexpression Huh7 cells compared with the control hepatoma cells (**Figure 3F**). Collectively, the above results indicated that miR-186 suppressed the self-renewal and tumorigenesis ability of liver CSCs.

Next, miR-186 sponge HCC cells were infected with miR-186 mimic virus (**Supplementary Figure 2A**). As shown in **Supplementary Figures 2B, C**, the upregulation of CSCs markers and transcription factors in miR-186 knockdown HCC cells could be reversed by ectopically overexpressed miR-186 in HCC cells. Moreover, the enhanced self-renewal ability and proportion of CSCs in miR-186 knockdown HCC cells were also impaired by ectopically overexpressed miR-186 in HCC cells (**Supplementary Figures 2D, E**). Taken together, these data further demonstrated that miR-186 inhibited the self-renewal and tumorigenesis ability of liver CSCs.

### PTPN11 Is Required for miR-186-Mediated Liver Cancer Stem Cell Expansion

**Figure 4:**
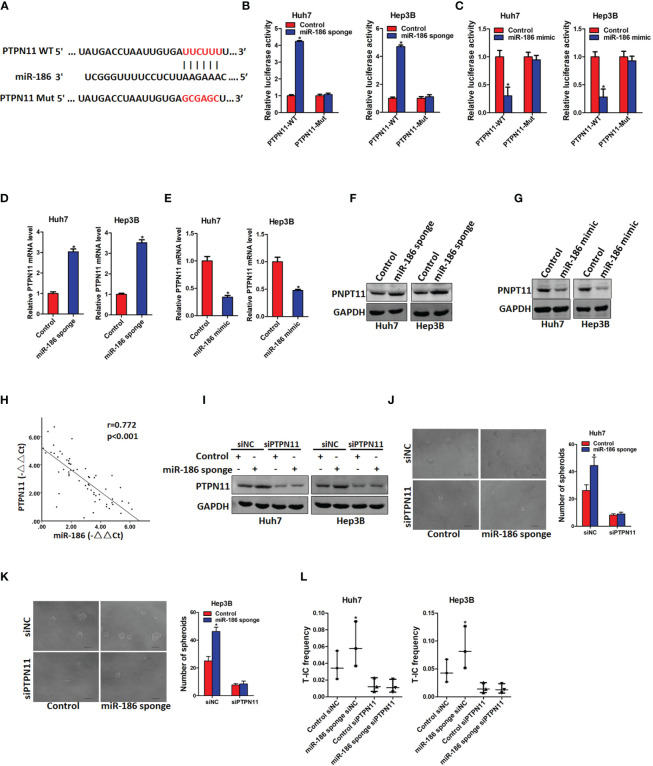
PTPN11 is required miR-186 mediated liver CSCs expansion. **(A)** PTPN11 3’UTR binding sites of miR-186 and its mutation counterpart were predicted by Targetscan and miRanda algorithms. **(B)** Wild-type and mutant PTPN11 3’UTR plasmids were transfected into miR-186 knockdown cells and control HCC cells, and luciferase activity was detected 24 h later. **(C)** The mRNA expression of PTPN11 in miR-186 knockdown cells and control HCC cells was checked by real-time PCR analysis. **(D)** The mRNA expression of PTPN11 in miR-186 overexpression cells and control HCC cells was checked by real-time PCR analysis, **(E)** Western blot was used to detect the protein expression of PTPN11 in miR-186 knockdown cells and control HCC cells. **(F)** Western blot was used to detect the protein expression of PTPN11 in miR-186 overexpression cells and control HCC cells. **(G)** The correlation between miR-186 and PTPN11 was analyzed in 60 HCC tissue samples. **(H)** miR-186 knockdown cells and control HCC cells were transfected with siPTPN11 and control siNC, and interference effect was detected by Western blot analysis. **(I)** miR-186 knockdown cells and control HCC cells were transfected with siPTPN11 and control siNC and were then inoculated into 96-well low adhesion plate to observe the spheroid formation. **(J, K)** miR-186 knockdown cells and control HCC cells were transfected with siPTPN11 and control siNC, and different concentrations of cells were inoculated into 96-well low adhesion plate, and spheroid formation was observed 7 days later. **(L)** miR-186 knockdown cells and control HCC cells were transfected with siPTPN11 and control siNC, gradient concentrations of HCC cells were seeded in low adhesion 96-well plate, and spheroids formation in each group was observed 7 days later, and the proportion of CSCs was counted and calculated. "*" means p<0.05. There was a statistical difference.

Next, we explore the underlying molecular that are involved in miR-186-mediated liver CSCs expansion. Targetscan and miRanda algorithms were used to predict the potential targets of miR-186. PTPN11 was found to be a potential binding site of miR-186 (**Figure 4A**). To test this hypothesis, wild-type and mutant reporter gene plasmids of PTPN11 was constructed and transfected into miR-186 overexpression or miR-186 knockdown and their corresponding control cells. It was found that luciferase activity was upregulated by interference of miR-186 in reporter gene construction containing wild-type 3’UTR, but not in construction containing mutant 3’UTR (**Figure 4B**). Conversely, the luciferase activity was downregulated by overexpression of miR-186 in reporter gene construction containing wild-type 3’UTR, but not in construction containing mutant 3’UTR (**Figure 4C**). The PTPN11 mRNA and protein expression were increased in miR-186 knockdown liver CSCs and decreased in miR-186 overexpression liver CSCs (**Figures 4D–G**). Correlation between the miR-186 and PTPN11 expression in 60 clinical liver cancer samples was analyzed and a significant negative correlation was deduced (r=0.772, p<0.005) (**Figure 4H**). These results suggest that miR-186 can directly regulates PTPN11 expression *via* its 3’UTR in liver CSCs.

Previous study reported that PTPN11 was upregulated in liver CSCs and promoted liver CSCs expansion (22). In order to explore whether miR-186 can regulate liver CSCs expansion *via* regulating PTPN11, siRNA of PTPN11 was transfected in miR-186 sponge HCC cells and control cells (**Figure 4I**). Spheroid formation assay showed that interference of PTPN11 abolished the discrepancy of self-renewal ability between miR-186 sponge HCC cells and control cells (**Figure 4J**). *In vitro* limiting dilution assay showed that interference of PTPN11 abrogated the discrepancy of CSCs proportion between miR-186 sponge HCC cells and control cells (**Figure 4K**). Taken together, these results demonstrated that miR-186 suppress the self-renewal and tumorigenicity of liver CSCs by downregulating PTPN11.

### miR-186 Overexpression Hepatocellular Carcinoma Are More Sensitive to Cisplatin Treatment

**Figure 5:**
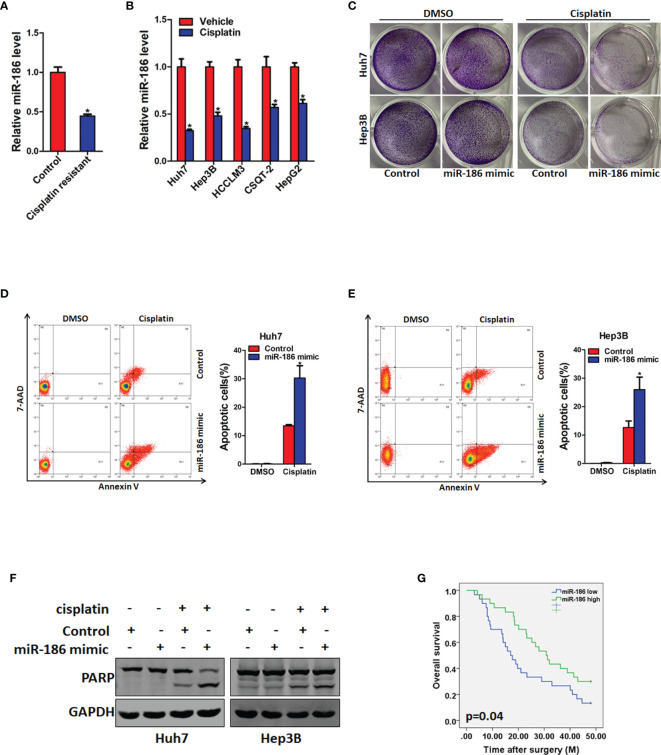
miR-186 overexpression HCC cells are more sensitive to cisplatin treatment. **(A)** The expression of miR-186 in cisplatin resistant PDX tissues and untreated PDX tissues was detected by real-time PCR analysis. **(B)** The expression of miR-186 in cisplatin resistant and untreated HCC cell lines was checked by real-time PCR analysis. **(C)** miR-186 knockdown cells and control HCC cells were seeded in 12-well plate, and treated with cisplatin (1 μg/L) for 7 days. The colony growth was examined. **(D)** Huh7 miR-186 knockdown and control HCC cells were seeded in 6-well plate, and treated with cisplatin (4 μg/L) for 48 h. Flow cytometry was used to detect the proportion of apoptotic cells. **(E)** Hep3B miR-186 knockdown and control HCC cells were seeded in 6-well plate, and treated with cisplatin (4 μg/L) for 48 h. Flow cytometry was used to detect the proportion of apoptotic cells. **(F)** miR-186 knockdown cells and control HCC cells were seeded in 6-well plate, and treated with cisplatin (4 μg/L) for 48 h after cell attachment. The expression of cleaved PARP is detected by Western blot analysis. **(G)** Prognosis analysis of miR-186 expression level in patients with postoperative TACE (miR-186 Low=30, miR-186 high=30). "*" means p<0.05. There was a statistical difference.

Numerous studies reported that CSCs were involved in the regulation of chemo-resistant of cancers (23). Therefore, we explore whether miR-186 is also participated in chemo-resistance of HCC. Cisplatin resistance xenograft and HCC cell lines were established as previous described (22). As expected, miR-186 expression was reduced in cisplatin resistant patient derived xenografts (PDX) tissues compared with untreated tissues (**Figure 5A**). The expression of miR-186 was also dramatically decreased in cisplatin resistant HCC cell lines compared with control cells (**Figure 5B**). Next, miR-186 overexpression HCC cells and control cells were treated with the same dose cisplatin for 7 days, and proliferation ability was detected by colony formation. It was found that miR-186 overexpression led to the sensitive of HCC cells to cisplatin-induced growth inhibition (**Figure 5C**). miR-186 overexpression HCC cells and control cells were treated with the same dose of cisplatin for 48 h, and apoptosis was detected by flow cytometry. It was found that the proportion of apoptotic cells in miR-186 overexpression group was significantly decreased (**Figures 5D, E**). Then cleaved PARP, protein marker of apoptosis, was found to be significantly higher in miR-186 overexpression HCC cells than that of the control cells under the same dose of cisplatin treatment (**Figure 5F**). Prognosis analysis of 60 patients with TACE after HCC surgery showed that the survival period was longer in patients with high expression of miR-186 than that of patients with low expression of miR-186 (**Figure 5G**), which indicated that HCC patients with high expression of miR-186 were more benefited from TACE.

## Discussion

Conventional radiation and chemotherapy, targeted drug and immunotherapy fail to eradicate tumors due to the existence of CSCs. However, the understanding of regulatory mechanisms for CSCs is limited. In the present study, we indicated the critical role of miR-186 in liver CSCs and the underlying mechanism. We also demonstrated the value of miR-186 to predict the TACE benefit for HCC patients.

Accumulating evidence proved that miRNAs are vital regulators of multiple proteins in various biological processes and tumor progression (24–26). Previous reports showed that miR‐186 plays complex roles in various malignancies. miR‐186 functions as an oncogene or tumor suppressor in tumorigenesis. It was upregulated in many types of cancers, including prostate cancer and nasopharyngeal carcinoma (27). Whereas it was also reduced in many types of cancer cells, such as liver cancer, cholangiocarcinoma and bladder cancer (28–30). However, the potential function of miR-186 in liver CSCs was unknown. In this study, our data showed that the expression of miR-186 was dramatically reduced in sorted CD133 or CD90 positive HCC cells compared with their control cells. We also found that miR-186 was downregulated in HCC spheres than adherent cells. We next examined the functional role of miR‐186 in liver CSCs expansion. miR-186 overexpression in HCC cells resulted in inhibition of liver CSCs self-renewal and tumorigenesis. Conversely, miR-186 knockdown in HCC cells enhanced the self-renewal and tumorigenesis capacity of liver CSCs.

PTPN11 belongs to the protein tyrosine phosphatase family and is the first oncogene that encodes phosphatase (31). Previous reports showed that PTPN11 worked as oncogene in liver cancer initiation (32). Whereas it was upregulated in HCC patients’ tissues and promoted HCC proliferation and metastasis (33). PTPN11 was also reported to play a pro-survival role in trophoblast stem cells, and homozygous inactivation of PTPN11 led to early embryonic lethality in mice (34). Deletion of PTPN11 also caused the aberrant differentiation and death of neural stem cells (35); in contrast, PTPN11 deficiency enhanced the self-renewal of embryonic stem cells (36). Xiang etc. reported that PTPN11 was upregulated liver CSCs and promoted liver CSC expansion *via* augmenting β-catenin pathway. However, the upstream of PTPN11 in liver CSCs was unclear. In this study, interference of miR-186 upregulated expression of PTPN11 mRNA and protein in liver CSCs. Enforced miR-186 expression decreased expression of PTPN11 mRNA and protein in liver CSCs. The negative correlation was confirmed between miR-186 and PTPN11 in liver cancer tissue samples. In addition, we found that miR-186 regulated the expression of PTPN11 by directly binding to its 3’-UTR. Further study showed that interference of PTPN11 can abolished the discrepancy between miR-186 mimic and control HCC cells in self-renewal and the proportion of CSCs. These results demonstrated that miR-186 directly target PTPN11 in liver CSCs.

Previous study showed that low PTPN11 expression exhibited superior response to TACE treatment following, but patients with high PTPN11 levels showed no response (33). Then we wondering whether miR-186 expression also predicts the TACE response in HCC patients. Herein, we observed that miR-186 expression was downregulated in cisplatin resistance xenograft and HCC cell lines. miR-186 overexpression in HCC cells resulted more sensitive of cisplatin induced HCC cells growth inhibition and apoptosis. Clinical investigation revealed that HCC patients with high miR-186 expression benefited from TACE administration after surgery, while patients with high miR-186 levels did not, which further indicates that miR-186 could serve as a biomarker in personalized therapy for HCC. Taken together, we found that miR-186 suppresses the expansion of liver CSCs by directly targeting PTPN11 and may serve as an optimal target in liver CSC-targeted therapy. The significance of miR-186 in HCC personalized medicine is worthy of further investigation.

In conclusion, we identified that miR-186 was downregulated in liver CSCs and inhibited liver CSCs self-renewal and tumorigenesis *via* directly targeting PTPN11. We also demonstrated the miR-186 to predict the TACE benefit for HCC patients. These findings of the present study not only shed a new light on the mechanism of liver CSCs but a potential therapeutic target against HCC.

## Data Availability Statement

The raw data supporting the conclusions of this article will be made available by the authors, without undue reservation.

## Ethics Statement

The studies involving human participants were reviewed and approved by the Ethics Committee of Eastern Hepatobiliary Surgery Hospital. The patients/participants provided their written informed consent to participate in this study. The animal study was reviewed and approved by The First Hospital of Jilin University.

## Author Contributions

HY, ZY, and YL conducted all the experiments and analyzed the data. PY provided the clinical samples, provided pathology evaluation, and analyzed the clinical data. JH provided support on the experimental techniques. HY wrote the manuscript. WJ and SC contributed to the revision. WJ and SC conceived the project and supervised all the experiments. All authors contributed to the article and approved the submitted version.

## Funding

This work was supported by the grant from the National Natural Science Foundation of China (81902942).

## Conflict of Interest

The authors declare that the research was conducted in the absence of any commercial or financial relationships that could be construed as a potential conflict of interest.
