# Supplementary material for: miR-186 Inhibits Liver Cancer Stem Cells Expansion via Targeting PTPN11
Source: Front Oncol. 2021 Mar 18;11:632976. doi: 10.3389/fonc.2021.632976 (PMC8012905; doi:10.3389/fonc.2021.632976)
Supplement: Supplementary file 1 [file DataSheet_1.pdf]

miR-186 Inhibits Liver Cancer Stem Cells Expansion via Targeting PTPN11

Haochen Yao[1,2,] Ziting Yang[3], Yan Lou[4], Juanjuan Huang[2], Pinghua Yang[5], Weiqi Jiang[5, *], Shuai Chen[1, *]

[1]Department of Emergency Surgery, The First Hospital of Jilin University, Changchun, China.
[2]Department of Pathogenobiology, The Key Laboratory of Zoonosis, Chinese Ministry of Education, College of Basic Medical Science, Jilin University, Changchun, China.
[3]The 964th hospital of the Chinese people's liberation Army, Changchun, Jilin Province, China.
[4]Department of Nephrology, the Second Hospital of Jilin University, Jilin, China.
[5]Department of Hepatic Surgery, Third Affiliated Hospital of Second Military Medical University, Shanghai, China.

***Correspondence author address:** Department of Emergency Surgery, The First Hospital of Jilin University, Changchun, China. E-mail addresses: qiweijiamg@sina.com (Weiqi Jiang); cosmos@jlu.edu.cn (Shuai Chen).

Running title: miR-186 suppresses liver CSCS expansion.

Funding information: This work was supported by the grand from the National Natural Science Foundation of China (81902942).

Supplementary Figure 1

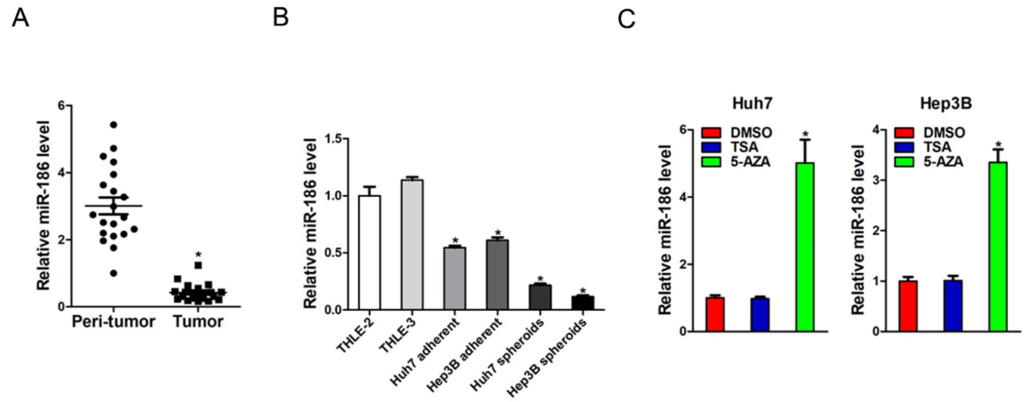

Supplementary Figure 1:

A. miR-186 expression in 20 pairs of HCC tumor and paired non-tumorous tissues was assessed by real-time PCR analysis.

B. miR-186 expression in normal hepatocytes, HCC cells and liver CSCs was checked by real-time PCR analysis.

C. HCC cells were treated with TAS (2 μM) or 5-AZA (5 μM) for 48 hours and then subjected to real-time PCR analysis.

Supplementary Figure 2

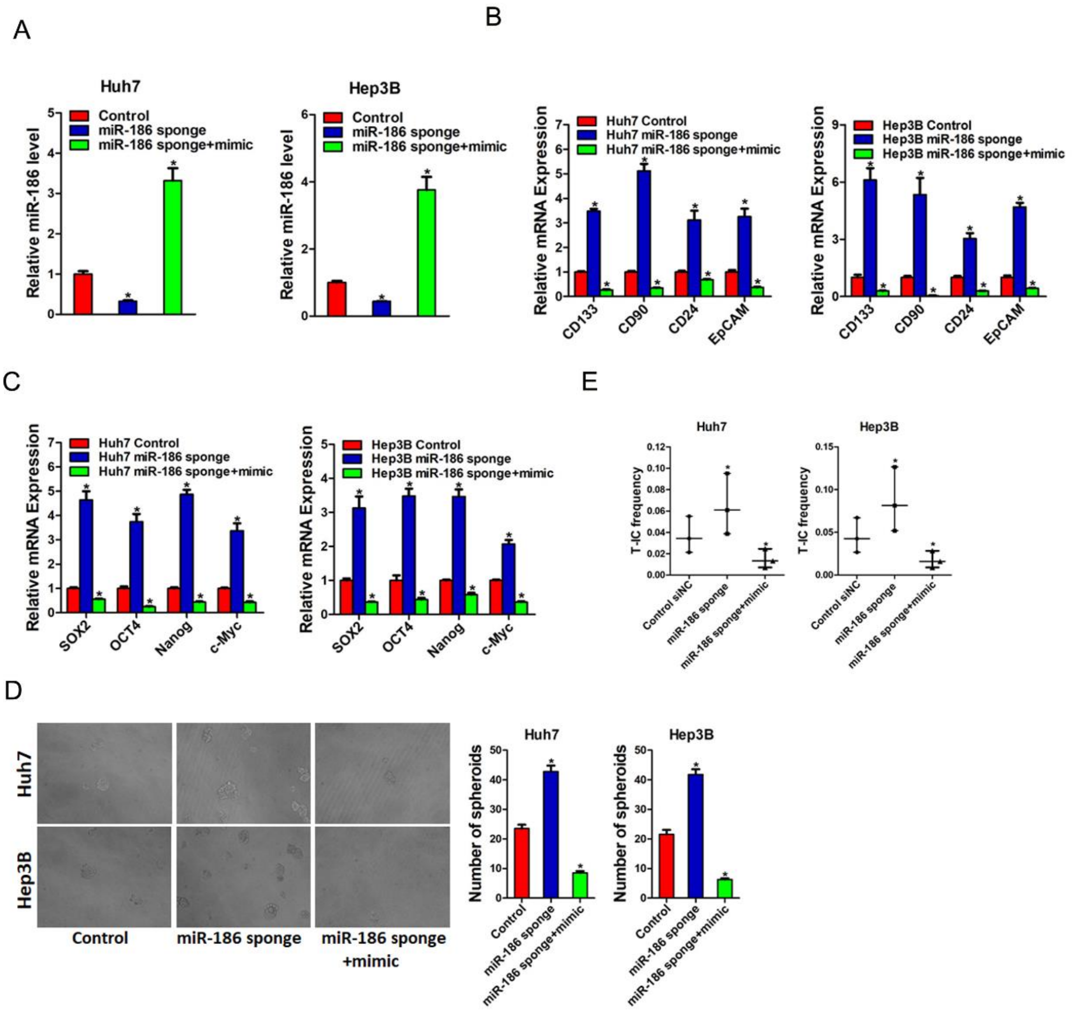

Supplementary Figure 2:

A. miR-186 sponge HCC cells were infected with miR-186 mimic virus and then subjected to real-time PCR analysis;

B. The expression of CSC markers CD133, CD90, CD24 and EpCAM in indicated HCC cells was determined by real-time PCR analysis;

C. The expression of transcription factors Sox2, Oct4, Nanog and c-Myc in indicated HCC cells was detected by real-time PCR analysis;

D. The indicated HCC cells were seeded in low adhesion 96-well plate, and spheroids were counted and photographed after 7 days;

E. Gradient concentrations of indicated HCC cells were seeded in low adhesion 96-well plate, and spheroids formation in each group was observed 7 days later, and the proportion of CSCs was counted and calculated.

Supplementary Tables

Supplementary Table 1. Clinicopathological features of 60 HCC patients

Characteristics		Total n=60
Age(year)	≤50	39
	>50	21
Gender	Male	53
	Female	7
HBsAg	Positive	54
	Negative	6
AFP(μg/L)	≤400	32
	>400	28
Tumor size(cm)	≤5	15
	>5	45
Tumor number	Single	37
	Multiple	23
Portal vein tumor thrombus	Yes	15
	No	45
iMVI	Yes	20
	No	30
BCLC stage	A	26
	B or C	34
TNM	I-II	27
	I-II	33

HBsAg: hepatitis B virus surface antigen; AFP: α-fetoprotein; TNM: Tumor-Nodes-Metastasis; BCLC: Barcelona Clinic Liver Cancer Staging.

Supplementary Table 2. Primer List.

Gene	Forward primer	Reverse primer (5'-3')
β-actin(Human)	Forward (5′- 3′)	GGCCCAGAATGCAGTTCGCCTT
	Reverse (5′- 3′)	AATGGCACCCTGCTCACGCA
CD133(Human)	Forward (5′- 3′)	AGAGGAAGCCGCAAC
	Reverse (5′- 3′)	CTGGCTCGTGAATTATTTAT
CD24(Human)	Forward (5′- 3′)	GCAAACAGATGTGTTCTTAAT
	Reverse (5′- 3′)	TCATCCCTAAGATCAAGTTT
CD90(Human)	Forward (5′- 3′)	GAATACGGAAATGGATTAAG
	Reverse (5′- 3′)	GTATTCATTTCCTCTGGTCT
EpCAM(Human)	Forward (5′- 3′)	CGCAGCTCAGGAAGAATGTG
	Reverse (5′- 3′)	TGAAGTACACTGGCATTGACGA
SOX2(Human)	Forward (5′- 3′)	TGGAGAAGGAATGGTCCACTTC
	Reverse (5′- 3′)	GGATAAGTACACGCTGCCCG
OCT4(Human)	Forward (5′- 3′)	ATGTGCGCGTAACTGTCCAT
	Reverse (5′- 3′)	CTGCAGTGTGGGTTTCGGGCA
c-Myc(Human)	Forward (5′- 3′)	CCCTCCACTCGGAAGGACTA
	Reverse (5′- 3′)	GCTGGTGCATTTTCGGTTGT
Nanog(Human)	Forward (5′- 3′)	AATACCTCAGCCTCCAGCAGATG
	Reverse (5′- 3′)	TGCGTCACACCATTGCTATTCTTC
U6(Human)	Forward	CTCGCTTCGGCAGCACATA
	Reverse	AACGATTCACGAATTTGCGT
PTPN11 (Human)	Forward	CTGCCTCCACACCAGTGATA
	Reverse	GGAGCCTGAGCAAGGAGC

Supplementary Table 3. Antibody List.

Antigens	Manufacturer	Application
PTPN11	Protientech, China	1:1000 for WB
PARP	Cell Signaling Technology, Beverly, MA	1:500 for WB
GAPDH	Santa Cruz Biotechnology, CA	1:5000 for WB
